# Management of Diabetes Mellitus and Hypertension During Hospitalization in Maxillofacial Departments

**DOI:** 10.3390/medicina61040712

**Published:** 2025-04-12

**Authors:** Andrei Krasovsky, Tal Capucha, Ragda Abdalla-Aslan, Nidal Zeineh, Amir Bilder, Ahmad Hija, Ori Blanc, Omri Emodi, Adi Rachmiel, Dekel Shilo

**Affiliations:** 1 Oral and Maxillofacial Surgery, Rambam Medical Care Campus, HaAliya HaShniya St 8, Haifa 3109601, Israel; capuchatal@gmail.com (T.C.); ragdaa@gmail.com (R.A.-A.); amirbilder@gmail.com (A.B.); hhija14@gmail.com (A.H.); oriblanc@gmail.com (O.B.); omri.emodi@gmail.com (O.E.); a_rachmiel@rambam.health.gov.il (A.R.); dekelshi@yahoo.com (D.S.); 2 Ruth & Bruce Rappaport Faculty of Medicine, Technion-Israel Institute of Technology, Haifa 3200003, Israel

**Keywords:** systemic, hypertension, diabetes mellitus, maxillofacial, hospitalization

## Abstract

*Background and Objectives*: Diabetes mellitus (DM) and hypertension (HTN) continue to increase and pose a significant burden on the health system worldwide. These patients comprise a significant portion of the hospitalized Oral and Maxillofacial Surgery (OMFS) department. Addressing and controlling DM and HTN during hospitalization should thus be one of the treatment goals. This study aims to investigate the management and outcomes of DM and HTN during the hospitalization period in the OMFS department. *Materials and Methods*: A retrospective analysis of patients with community-diagnosed DM and/or HTN admitted to the OMFS department between 2017 and 2019 was conducted at Rambam Healthcare Campus, Haifa, Israel. Linear regression analysis was used to determine trends in changes in blood pressure and blood glucose levels during hospitalization. The impact of consults from internal medicine experts and endocrinologists was tested using a paired-samples *t*-test. *Results*: A statistically significant reduction was observed in all systolic HTN patients categorized as stages 1 and 2, *p* = 0.012 and *p* = 0.001, respectively. A statistically significant (*p* = 0.012) reduction in blood glucose levels in all DM patients with initial values higher than 250 mg/dL was observed. A statistically significant reduction in blood glucose levels was observed in the DM group of patients who received endocrinologist consultations (*p* = 0.012). *Conclusions*: Addressing patients with systemic conditions during hospitalization in the OMFS department is mandatory. External medical consulting can be of great value for short-term treatment during hospitalization and may also have long-term benefits after discharge to the community. The main limitations of this study include retrospective data acquisition, a small sample size, and a lack of data regarding the impact of pain management on blood pressure and glucose levels.

## 1. Introduction

The prevalence and incidence of diabetes mellitus (DM) have been steadily on the rise over the past three decades [1]. The worldwide prevalence of hypertension (HTN) exceeds 1.3 billion, and by 2025, the number is predicted to increase to 1.56 billion [2]. HTN and DM are often linked and occur together as both are aspects of metabolic syndrome [3]. HTN, and especially DM, were thoroughly studied in the context of orthopedic surgeries. In orthopedic surgical patients, DM is associated with higher rates of perioperative complications, such as pneumonia, surgical site infection, blood transfusion, reoperation, extended hospital length of stay, and in-hospital mortality [4,5]. HTN has been identified as an independent risk factor for surgical site infections, potentially leading to a longer duration of hospital stay [6]. HTN may also promote deep vein thrombosis (DVT) following orthopedic surgeries, and may be an essential risk factor for DVT occurrence [7]. HTN treatment is usually achieved with relatively simple and cost-effective medical management. Orthopedic surgeons are encouraged to adopt a multi-disciplinary approach to managing hypertension, particularly in older patients, to decrease the length of stay and postoperative complications [8]. Similarly, DM patients also require consideration when planning orthopedic surgeries. Care improvement with patient benefits and cost savings is possible in these patients [9]. Hypotensive anesthesia is commonly applied in OMFS, which requires a bloodless operating field to reduce bleeding and to allow easier identification of the anatomical structures [10]. According to a recent comprehensive review in the field of OMFS [11], the most vulnerable patients to hypoperfusion in the perioperative setting are older patients with isolated systolic hypertension. Also, the same publication indicated a double risk for postoperative infection in patients with hyperglycemia, specifically for patients undergoing oral and maxillofacial procedures. A multidisciplinary approach has become increasingly popular in medicine, enabling more accurate evaluation and targeted treatment adjustments in patients with complex comorbidities. It is common practice for hospitals to provide consultations from various relevant medical specialists. During hospitalization, internal medicine specialists and endocrinologists may provide valuable input for patients with HTN and DM. Furthermore, patients receiving treatment in the community may not consistently follow the prescribed treatment plans and may not regularly visit their physicians, potentially resulting in poorly managed diseases. Therefore, addressing patients’ other medical conditions during hospitalization may provide additional value.

Elderly medically compromised patients are commonly referred and treated in the Oral and Maxillofacial Surgery (OMFS) departments [12]. It is well known that comorbidities affect OMFS outcomes and prolong hospital stays [13]. However, to our knowledge, no studies exist regarding the efficacy of addressing systemic disorders, such as HTN and DM, in patients hospitalized in the OMFS department and how the hospitalization period affects these patients’ comorbidities. Inspired by the available orthopedic data, we aim to investigate the impact of hospitalization in the OMFS department on the conditions of HTN and DM in systemic patients. In this work, we aim to investigate the effectiveness of managing DM and HTN in patients hospitalized in OMFS departments through external medical consultations with endocrinologists and internal medicine experts.

## 2. Materials and Methods

Data from patients treated in the OMFS department at Rambam Healthcare Campus, Haifa, Israel, between 2017 and 2019 were retrospectively collected from electronic medical records. Demography, medical history, cause of hospitalization, type of surgical intervention, and the number of medical consultations given by experts outside the OMFS field were included.

Enrollment criteria were: (1) age equal or above 50 years old; (2) hospitalized for surgical intervention, observation, or medical examination at the department of OMFS between 2017 and 2019; (3) community-diagnosed systemic condition of DM, HTN, or both; (4) available data of glucose levels, systolic and diastolic blood pressure measurements during the hospitalization period. Exclusion criteria were: (1) patients under 50 years old; (2) patients without accessible medical history (e.g., tourists and/or foreign workers).

Patients were divided into four main groups: (1) patients with HTN who were given an internal expert consult regarding their blood pressure during the hospitalization period; (2) patients with HTN who did not receive an internal expert consult despite high blood pressure recordings during hospitalization period; (3) patients with DM who were given an endocrinologist expert consult regarding their high glucose levels during the hospitalization period; (4) patient with DM who did not receive an endocrinologist expert consult despite their high glucose levels during the hospitalization period. The consultation waiting time during hospitalization was also recorded.

SPSS (Version 20; IBM, Armonk, NY, USA) software was used for statistical analysis. A difference with a *p*-value of less than 0.05 was considered statistically significant. For each of the four patient groups, a linear regression line was constructed using data on blood pressure and nonfasting glucose levels recorded during the hospitalization period. Using the linear regression equation (y = ax + b) for each group of patients, the initial (A) and final (B) virtual values of blood pressure and glucose levels were defined. A paired-samples *t*-test was conducted to identify if statistically significant changes exist between initial and final recordings of systolic and diastolic blood pressure and blood glucose levels during hospitalization. Furthermore, blood pressure and glucose levels were categorized to investigate potential relationships between experts’ consultations and changes in blood pressure and glucose levels recorded during hospitalization, using the chi-square test for independence. Systolic blood pressure was categorized as follows: 0—normal < 120 mmHg; 1—prehypertension—120–139 mmHg; 2—stage 1 hypertension—140–159 mmHg; 3—stage 2 hypertension ≥ 160 mmHg. Diastolic blood pressure was categorized as follows: 0—normal < 80 mmHg; 1—prehypertension—80–89 mmHg; 2—stage 1 hypertension—90–99 mmHg; 3—stage 2 hypertension ≥ 100 mmHg. Blood glucose was categorized as follows: 0 < 100 mg/dL; 1—100–180 mg/dL; 2—181–250 mg/dL; 3 > 250 mg/dL. Each of the above categories was also analyzed using a paired-samples *t*-test to determine if there was a statistically significant change between the initial and final value during the hospitalization period.

## 3. Results

This study included 235 patients with HTN, DM, or both (Table 1). A total of 154 patients were in the HTN group (88 males, 66 females), with an average age of 69 and a mean hospitalization time of 5.1 days. A total of 81 patients were in the DM group (42 males, 32 females), with an average age of 66 and a mean hospitalization time of 5.7 days. A total of 55 patients suffered from both HTN and DM, while 26 patients suffered from DM only, and 99 patients suffered from HTN only. In the HTN group, the distribution of etiology for hospitalization was: trauma—26 (22.4%), abscess, cellulitis—19 (16.4%), osteomyelitis, MRONJ, osteoradionecrosis—11 (9.5%), salivary glands—14 (12.1%), malignancy—11 (9.5%), benign tumors, teeth extraction, implantology—25 (21.6%), observation, medical examination—8 (6.9%), and other—2 (1.7%). In the DM group, the distribution of etiology for hospitalization was: trauma—16 (19.8%), abscess, cellulitis—19 (23.5%), osteomyelitis, MRONJ, osteoradionecrosis—4 (4.9%), salivary glands—8 (9.9%), malignancy—11 (13.6%), benign tumors, teeth extraction, implantology—16 (19.8%), observation, medical examination—5 (6.2%), and other—2 (2.5%).

The distribution of surgical interventions in the HTN group was: no surgery and no antibiotic treatment—11 (9.5%), only antibiotic treatment—16 (13.8%), major surgery (conducted under general anesthesia and ventilation)—49 (42.2%), minor surgery (no ventilation)—29 (25.0%), and salivary glands surgery—11 (9.5%). The distribution of surgical interventions in the DM group was: no surgery and no antibiotic treatment—5 (6.2%), only antibiotic treatment—13 (16.0%), major surgery (conducted under general anesthesia and ventilation)—33 (40.7%), minor surgery (no ventilation)—24 (29.6%), and salivary glands surgery—6 (7.4%).

**Figure 1:**
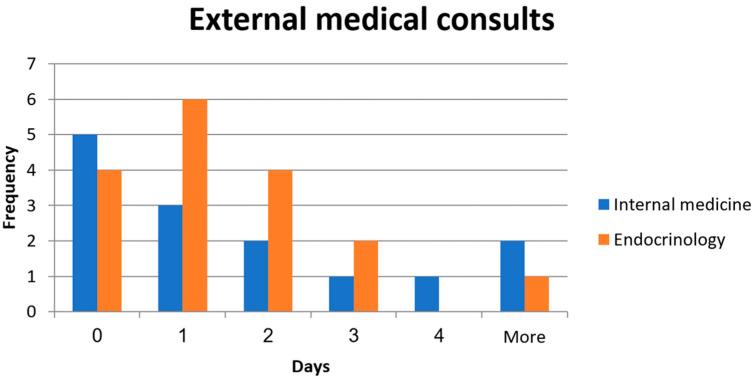
Time histogram (days) of external medical consults for HTN (by internal medicine expert) and DM (by endocrinologist) since hospitalization and until the consult was delivered.

Internal medicine experts provided consultations for 14 patients, while endocrinologists provided 17 consultations (Table 2). Five patients in each group were given more than one expert consultation. Most patients in each group required a change in their medication regimen following the consultation. The average consultation waiting time was less than a day. The average time for consulting since hospitalization was more than a day. Most consultations were delivered within the first two days after hospitalization (Figure 1).

Further, two consults of experts in internal medicine were given 8 and 23 days after hospitalization. One consult with an endocrinologist was given six days after the first day of hospitalization.

Table 3 presents linear regression statistics for each of the four research groups. Based on linear regression parameters (Table 4), a statistically significant reduction in blood pressure was observed in the systolic and diastolic HTN groups of patients who did not receive internal medicine expert consultations, *p* = 0.005 and *p* = 0.002, respectively. Additionally, a statistically significant reduction in blood glucose levels was observed in the DM group of patients who received consultations from an endocrinologist (*p* = 0.012).

Table 5 presents the distribution of blood pressure and glucose levels categories. A chi-square test of independence examined the relation between categories of average systolic and diastolic blood pressures and consults given by internal medicine experts. A significant association (*p* = 0.006, Cramer’s V = 0.325) was found between average systolic blood pressure categories on the first day of hospitalization and a consult given by an internal medicine expert. Patients hospitalized in the OMFS department with high systolic blood pressure were more likely to have a consult visit by an internal medicine expert. No significant association was found in diastolic blood pressure (*p* = 0.505). A chi-square test of independence examined the relation between categories of average blood glucose and consults given by endocrinologists. The relationship between these variables was significant (*p* = 0.000043, Cramer’s V = 0.522). Patients hospitalized in the OMFS department with high blood glucose levels were more likely to have a consult visit by an endocrinologist.

A statistically significant reduction was observed in all systolic HTN patients categorized as stages 1 and 2, *p* = 0.012 and *p* = 0.001, respectively (Table 6). Table 7 presents a statistically significant (*p* = 0.012) reduction in blood glucose levels in all DM patients with initial values greater than 250 mg/dL.

## 4. Discussion

According to the literature [14], the highest number of HTN and DM is found in the age group of 40–60. Hence, we decided to include patients in the age groups equal to or above 50 years old for a more statistically representative and homogenous study group. Investigating patients with HTN and DM is crucial because both are commonly hospitalized in the OMFS department due to various etiologies. This statement is supported by the data from this study, indicating patients with HTN and DM were hospitalized in the OMFS department due to trauma, odontogenic infections, osteomyelitis, malignant and benign tumors, and salivary gland diseases. Patients with HTN and DM are also in considerable demand for dental extractions and implant therapies. Also, HTN and DM patients are usually treated with different combinations of medication classes and require regular follow-ups with community physicians and occasional treatment modifications, all of which can reduce patients’ compliance and lead to a poorly controlled disease. As a result, their conditions may not be well-managed and could worsen during the stress of hospitalization. As shown in Table 5, the majority (44.8%) of HTN patients admitted to this study were poorly controlled and categorized as stage 1 HTN, while 13.6% had stage 2 HTN, together comprising more than half of the patients with uncontrolled blood pressure.

Endocrine Society and the ADA/AACE Practice Guidelines recommend <180 mg/dL of random glucose for most non-critically ill patients treated with insulin [15]. The American Diabetes Association has suggested that most general medicine and surgery patients in non-ICU settings target glucose between 140 and 180 mg/dL [16]. In our study, 37% of the DM patients on admission had nonfasting blood glucose levels above 180 mg/dL, indicating a poorly controlled glycemic index. To conclude, a relatively high percentage of patients diagnosed with HTN and/or DM who were hospitalized in the OMFS department were also in need of systemic workup and management. As indicated in Table 2, most consultations resulted in recommendations for adjusting medications related to systemic conditions. This highlights the importance of having internal medicine specialists and endocrinologists involved in managing hospitalized patients with HTN and DM in the OMFS department. It also highlights the importance of pertinent clinical knowledge for oral and maxillofacial surgeons in recognizing and addressing significant systemic issues in patients. Most consultations were provided on the same day or the next day they were ordered (Figure 1), demonstrating efficient collaboration within the hospital environment. We believe that this should be the gold standard in treating systemic patients.

Surprisingly, the group of patients who did not receive internal medicine expert consultations and had no changes in medication regimens showed a significant reduction in systolic and diastolic blood pressure values during hospitalization. In contrast, the values of the consulted group did not change significantly (Table 4). This unexpected result can be explained by the fact that blood pressure normalization may require a lengthy process until the most effective medical treatment is tailored for the patient. The medical changes implemented may not immediately impact blood pressure and may require further adaptations by the community physician after discharge. It is known that pain can elevate blood pressure [17]. Patients admitted to the OMFS department often experience varying levels of pain before and after undergoing surgical intervention. This work did not study the potential contribution of pain specialists to managing high blood pressure in this context, which is one of this study’s limitations. When all HTN patients were analyzed as a single group, a significant reduction in systolic and diastolic blood pressure levels was found in patients with higher initial values (Table 6). Improving the overall patient condition due to a surgical intervention may explain this general trend.

DM patient analysis produced more anticipated outcomes. Patients who received consultations with an endocrinologist experienced a significant reduction in high glucose levels (Table 4). A statistically significant decrease in blood glucose was also demonstrated in all DM patients with the highest initial levels. Again, the reduction in physiological stress due to surgical intervention and endocrinological modifications may have contributed to achieving a more balanced diabetic state.

This study’s limitations include retrospective data acquisition, a small sample size in some analyzed groups, and, as previously mentioned, the lack of data regarding the impact of pain management on blood pressure and glucose levels. Additionally, in this study, we did not perform a separate analysis for patients diagnosed with both HTN and DM, but included them in both the HTN and DM groups. In patients undergoing general surgery, postoperative complications are predominantly linked to comorbidities such as HTN and DM [18]. However, chronic kidney and pulmonary diseases are also common among general surgery patients and should be investigated in the OMFS setting [18]. Confounding factors such as age and other comorbidities were not addressed in this study.

In summary, addressing the systemic condition of patients hospitalized in OMFS departments and judiciously using external consultations provides significant value in the overall management of these patients. Normalizing blood pressure during hospitalization proved to be more challenging and less predictable than normalizing blood glucose levels. Despite this, systemic consultations may also influence patients’ behaviors after discharge and provide valuable information for ongoing treatment in the community; thus, they should be encouraged. Oral and maxillofacial surgeons must thoroughly review the medical history of hospitalized patients and closely monitor vital signs and laboratory test results. Undiagnosed or uncontrolled systemic conditions may be identified and normalized during the hospitalization period, and relevant recommendations may be given for further investigation in the community after discharge. Further research investigating larger sample sizes and the impact of other systemic conditions on OMFS hospitalized patients may be beneficial. Additionally, we strongly recommend expanding future research to include follow-up analysis of patients’ HTN and DM control, for a better understanding of the impact of hospital consultations on community management of chronic systemic conditions.

## 5. Conclusions

The main finding of this study revealed that blood glucose levels were more consistently normalized during the hospitalization period than blood pressure levels, and this normalization was associated with consultations from an endocrinologist. External medical consultations are highly recommended for patients with systemic conditions while hospitalized in OMFS departments.

## Figures and Tables

**Table 1:** Demographic and medical history.

Variables	DM	HTN
Gander, n (%)		
Male	42 (51.9)	88 (57.1)
Female	39 (48.1)	66 (42.9)
Total	81 (100)	154 (100)
Mean age, y (SD)	66 (9.4)	69 (10.8)
Mean HT, d (SD)	5.7 (4.5)	5.1 (4.1)

DM—diabetes mellitus, HTN—hypertension, HT—hospitalization time. y—years, d—days, and SD—Standard Deviation.

**Table 2:** External medical consulting.

Expert	Consult A	Consult B	Pharmacological Intervention	Time A, d (SD)	Time B, d (SD)
Internal medicine	14	5	10	0.36 (0.5)	3.21 (6.1)
Endocrinologist	17	5	15	0.12 (0.3)	1.5 (1.5)

Medical consultations that were provided by internal medicine experts for HTN and by endocrinologists for patients with DM. Two internal medicine expert consults regarding HTN were given to patients who were not classified as having HTN but as having DM. Consult A—number of patients given at least one consult; Consult B—number of patients given more than one consult; Pharmacological intervention—number of patients whose medications were changed as a result of the consultations (date and intervention data of one patient were missing); Time A—mean time in d (days) since consult was ordered and delivered; Time B—mean time in d (days) since the first day of hospitalization and until consult was delivered.

**Table 3:** Linear regression statistics.

	N	Multiple R Mean	R Square Mean	SE Mean	Measurements N Mean
HTN patients given Internal medicine expert consult					
Systolic	12	0.913	0.629	16.644	29.333
Diastolic	12	0.135	0.163	9.831	29.333
HTN patients not given Internal medicine expert consult					
Systolic	142	0.221	0.079	14.112	14.200
Diastolic	142	0.227	0.117	9.093	14.160
DM patients given endocrinologist expert consult					
Glucose	17	0.346	0.026	65.692	30.24
DM patients not given endocrinologist expert consult					
Glucose	64	0.292	0.195	34.472	12.69

A regression line was constructed to determine initial and final virtual values of systolic and diastolic blood pressures, and nonfasting glucose levels. N—number of patients; Multiple R—correlation coefficient; R square—coefficient determination; SE—Standard Error; Measurements N—The average number of times systolic/diastolic/glucose measures were recorded in each patient.

**Table 4:** Paired-samples t-test for comparing the mean initial and final virtual values of systolic and diastolic blood pressures, and nonfasting glucose levels.

	Mean (mmHg)	N	SDm	Mean Difference	SDmd	95% CI		Df	Sig (2-Tailed)
						Lower	Upper		
HTN patients given Internal medicine expert consult									
Systolic A	152.9	12	21.4	−2.0	23.1	−16.7	12.7	11	0.766
Systolic B	154.9		24.2						
Diastolic A	75.1		12.1	−3.1	8.9	−8.8	2.6	11	0.254
Diastolic B	78.2		10.9						
HTN patients not given Internal medicine expert consult									
Systolic A	138.7	142	14.6	3.8	15.9	1.1	6.4	141	0.005
Systolic B	134.9		15.5						
Diastolic A	75.3		10.8	2.7	12.9	0.4	4.7	141	0.002
Diastolic B	72.7		12.3						
	**Mean** **(mg/dL)**	**N**	**SDm**	**Mean Difference**	**SDmd**	**95% CI**		**Df**	**Sig (2-Tailed)**
						**Lower**	**Upper**		
DM patients given endocrinologist expert consult									
Glucose A	240.4	17	75.6	61.2	89.3	15.3	107.1	16	0.012
Glucose B	179.2		52.5						
DM patients not given endocrinologist expert consult									
Glucose A	158.1	64	47.7	13.0	53.5	−0.3	26.4	63	0.056
Glucose B	145.1		40.7						

A—starting value of regression line; B—end value of regression line; SDm—Standard Deviation of mean; SDmd—Standard Deviation of mean difference; CI—Confidence Interval; Df—degrees of freedom.

**Table 5:** Distribution of blood pressure and glucose measures categories.

		Frequency	
Category	Systolic (%)	Diastolic (%)	Glucose (%)
0	16 (10.4)	94 (61.0)	6 (7.4)
1	48 (31.2)	37 (24.0)	45 (55.6)
2	69 (44.8)	19 (12.3)	21 (25.9)
3	21 (13.6)	4 (2.6)	9 (11.1)

The average virtual values of systolic blood pressure, diastolic blood pressure, and blood glucose levels on the first day of hospitalization were calculated using linear regression and categorized. Systolic blood pressure measures were categorized as follows: 0—normal < 120 mmHg; 1—prehypertension—120–139 mmHg; 2—stage 1 hypertension—140–159 mmHg; 3—stage 2 hypertension ≥ 160 mmHg. Diastolic blood pressure measures were categorized as follows: 0—normal < 80 mmHg; 1—prehypertension—80–89 mmHg; 2—stage 1 hypertension—90–99 mmHg; 3—stage 2 hypertension ≥ 100 mmHg. Blood glucose measures were categorized as follows: 0 < 100 mg/dL; 1—100–180 mg/dL; 2—181–250 mg/dL; 3 > 250 mg/dL.

**Table 6:** Paired-samples *t*-test for comparing the mean initial and final virtual values of systolic and diastolic blood pressure levels in each category.

		Mean (mmHg)	N	SDm	Mean Difference	SDmd	95% CI		Df	Sig (2-Tailed)
							Lower	Upper		
Systolic blood pressure categories of all HTN patients										
<120	A	118.9	16	8.7	−6.3	13.4	−13.4	0.8	15	0.080
	B	125.2		13.8						
120–139	A	129.4	48	9.9	−0.9	12.9	−4.7	2.7	47	0.632
	B	130.3		13.8						
140–159	A	145.4	69	8.2	5.7	17.9	1.2	9.9	68	0.012
	B	139.8		17.8						
>159	A	161.0	21	12.7	12.9	15.6	5.7	20.0	20	0.001
	B	148.2		14.2						
Diastolic blood pressure categories of all HTN patients										
<80	A	68.9	94	7.9	−0.9	13.9	−3.7	1.9	93	0.527
	B	69.8		12.9						
80–89	A	83.0	37	3.7	6.8	9.1	3.7	9.8	36	0.00007
	B	76.3		9.4						
90–99	A	87.0	19	6.9	5.9	7.8	2.2	9.7	18	0.003
	B	81.0		8.15						
>99	A	97.9	4	8.3	12.3	12.4	−7.5	32.0	3	0.143
	B	85.7		4.5						

A—initial value of regression line; B—final value of regression line; SDm—Standard Deviation of mean; SDmd—Standard Deviation of mean difference; CI—Confidence Interval; Df—Degrees of freedom.

**Table 7:** Paired-samples *t*-test for comparing the mean initial and final virtual values of nonfasting glucose levels in each category.

		Mean (mg/dL)	N	SDm	Mean Difference	SDmd	95% CI		Df	Sig (2-Tailed)
							Lower	Upper		
Glucose levels categories of all DM patients										
<100	A	106.0	6	24.1	−5.8	27.1	−34.2	22.5	5	0.619
	B	111.9		12.7						
100–180	A	147.7	45	34.9	10.3	44.9	−3.2	23.8	44	0.131
	B	137.4		31.3						
181–250	A	209.1	21	49.3	27.5	77.8	−7.9	62.9	20	0.121
	B	181.6		45.0						
>250	A	281.2	9	63.7	96.5	89.5	27.7	165.3	8	0.012
	B	184.7		63.8						

A—initial value of regression line; B—final value of regression line; SDm—Standard Deviation of mean; SDmd—Standard Deviation of mean difference; CI—Confidence Interval; Df—Degrees of freedom.

## Data Availability

The raw data supporting the conclusions of this article will be made available by the authors on request.
